# Hypotension after Anesthesia Induction: Target-Controlled Infusion Versus Manual Anesthesia Induction of Propofol

**DOI:** 10.3390/jcm12165280

**Published:** 2023-08-14

**Authors:** Serap Aktas Yildirim, Lerzan Dogan, Zeynep Tugce Sarikaya, Halim Ulugol, Bulent Gucyetmez, Fevzi Toraman

**Affiliations:** 1 Department of Anesthesiology and Reanimation, Acibadem Mehmet Ali Aydinlar University School of Medicine, Istanbul 34752, Turkey; tugcesrky@gmail.com (Z.T.S.); halimulugol@yahoo.com.tr (H.U.); bulentgucyetmez@gmail.com (B.G.); ftoraman@gmail.com (F.T.); 2 Acibadem Altunizade Hospital, Istanbul 34662, Turkey; lredzheb@gmail.com

**Keywords:** post-induction hypotension, target-controlled infusion, propofol, cardiac power output, stroke volume index

## Abstract

Background: Post-induction hypotension frequently occurs and can lead to adverse outcomes. As target-controlled infusion (TCI) obviates the need to calculate the infusion rate manually and helps safer dosing with prompt titration of the drug using complex pharmacokinetic models, the use of TCI may provide a better hemodynamic profile during anesthesia induction. This study aimed to compare TCI versus manual induction and to determine the hemodynamic risk factors for post-induction hypotension. Methods: A total of 200 ASA grade 1–3 patients, aged 24 to 82 years, were recruited and randomly assigned to the TCI (n = 100) or manual induction groups (n = 100). Hemodynamic parameters were monitored with the pressure-recording analytic method. The propofol dosage was adjusted to keep the Bispectral Index between 40 and 60. Results: Post-induction hypotension was significantly higher in the manual induction group than in the TCI group (34% vs. 13%; *p* < 0.001, respectively). The propofol induction dose did not differ between the groups (TCI: 155 (135–180) mg; manual: 150 (120–200) mg; *p* = 0.719), but the induction time was significantly longer in the TCI group (47 (35–60) s vs. 150 (105–220) s; *p* < 0.001, respectively). In the multivariable Cox regression model, the presence of hypertension, stroke volume index (SVI), cardiac power output (CPO), and anesthesia induction method were found to predict post-induction hypotension (*p* = 0.032, *p* = 0.013, *p* = 0.024, and *p* = 0.015, respectively). Conclusion: TCI induction with propofol provided better hemodynamic stability than manual induction, and the presence of hypertension, a decrease in the pre-induction SVI, and the CPO could predict post-induction hypotension.

## 1. Introduction

Hemodynamic changes during the induction of anesthesia may have adverse outcomes and should therefore be avoided [1]. Although many studies have been conducted on this topic, approximately 20–30% of patients still develop post-induction hypotension [2]. A decrease in blood pressure during induction may occur due to multifactorial causes, such as the type and dose of the anesthetic agent used, a high American Society of Anesthesiologists (ASA) patient score, hypovolemia, or ventricular dysfunction [3]. Blood pressure during anesthesia induction with propofol can be decreased by venous dilatation, arterial dilatation, or a reduction in cardiac contractility, but the impact that different hemodynamic mechanisms and the induction method have on hypotension remains unclear [4,5].

Target-controlled infusion (TCI) systems aim to reach the theoretically targeted blood or brain concentration of anesthetic agents based on the patient’s age, weight, and height with computer-assisted algorithms [6]. In manual anesthesia induction, anesthetic agents are administered at a fixed dose and rate according to the patient’s weight, which may cause hypotension in patients with low cardiovascular performance [7]. As the TCI system obviates the need to calculate the infusion rate manually and helps to adjust the dosage to the needs of each patient, the use of TCI may provide a better hemodynamic profile during anesthesia induction [8].

TCI technology has been available for clinical use in anesthesia for approximately two decades in most countries. Currently, different pharmacokinetic TCI models (Marsh, Schnider, White, Eleveld, etc.) for propofol, targeting blood or effect-site concentrations, are commercially available. Typically, plasma or effect-site concentrations are targeted, but the plasma is not the site of the drug effect. The differences between these models in terms of drug doses given are not the same. These models use different patient covariates and rate constants. The implementation of the pharmacokinetic properties for TCI systems is mainly based on two different models: the Marsh model and the Schnider model. Regardless of the TCI model used, it is important to recognize the possibility of variations in pharmacokinetics and pharmacodynamics between different patients and the importance of adjusting the target concentrations to achieve the desired effect.

Different TCI models for propofol have been compared to evaluate the relevant pharmacological aspects, such as accuracy in concentration calculations, pharmacokinetic and pharmacodynamic properties, and safety [9]. Various modes of administration (e.g., manual infusions) with different types of general anesthesia and even different kinds of TCI models controlled by effect or plasma target concentrations have also been studied [10,11]. The most predominant adverse effect site in all of these studies was hemodynamic deterioration. Currently, one of the most widely used models for propofol with an effect site target is the Schnider model, through which a more stable induction can be achieved through its comparatively lower starting dose [12].

In the present study, we compared the hemodynamic effects of Bispectral Index™ (BIS)-guided manual propofol induction and propofol TCI induction (Schnider model) through hemodynamic parameters monitored by the pressure-recording analytical method (PRAM). The primary objective was to determine the effects of the two methods on the development of post-induction hypotension, with the secondary objective of identifying the hemodynamic parameters that could predict post-induction hypotension. We believed that examining the hemodynamic variables monitored with PRAM during these two induction methods would provide a better understanding of the mechanism of hypotension due to anesthesia induction.

## 2. Materials and Methods

### 2.1. Ethical Approval

This single-center, prospective, non-blinded, and randomized controlled trial was conducted between December 2022 and July 2023 at Acibadem Altunizade Hospital, which belongs to Acibadem MAA University. Ethical approval was obtained from the Regional Ethical Committee of Acibadem MAA University (ATADEK-2022-18/04). The study complied with the Declaration of Helsinki’s ethical principles for medical research involving human subjects. All patients provided their informed consent before the study. 

### 2.2. Trial Registration 

The trial was prospectively registered (NCT05708638) on 23 January 2023.

### 2.3. Patients

Patients with an ASA physical status 1–3, aged 24 to 82 years, and a weight range between 48 and 90 kg who were scheduled for elective major abdominal surgery (gynecologic surgery and gastrointestinal surgery) with intra-arterial blood pressure monitoring before induction were recruited. Exclusion criteria included allergy to any study drug, pregnancy, treatment with opiates, drug addiction, arrhythmia, intubation difficulty, severe valvular heart disease, severe pre-existing lung disease, morbid obesity, age under 18 years, epidural analgesia in combination with general anesthesia, and emergency surgery.

### 2.4. Randomization and Blinding

All patients who agreed to participate and met the inclusion criteria were randomly assigned to the TCI group or the manual anesthesia induction group through a 1:1 allocation. A total of 211 patients were recruited; of these, five patients were excluded due to their withdrawal of informed consent, and six patients were excluded because of missing data. A list of randomization numbers was prepared by a statistician, and the group assignment was kept in a sealed envelope that was opened by the attending anesthetist before the induction of anesthesia. Due to the nature of the intervention, neither the participants nor the researcher could be blinded to the allocation. The patients were informed of the group to which they had been randomly allocated. As a result, 200 patients were randomly assigned to have either TCI induction (100) or manual induction (100) of propofol for general anesthesia. 

### 2.5. Study Protocol

No premedication or sedative agent was given before induction. Ringer’s lactate solution at a rate of 5 mL/kg was started in all patients. Both groups of patients received a remifentanil infusion of 0.15 μg kg ^−1^ min^−1^ at the beginning of the anesthesia induction and a bolus dose of remifentanil of 1 mg kg^−1^ 30 s before intubation. The TCI pump used for propofol was the Orchestra^®^ Base Primea (Fresenius-Kabi, India). Patients who were assigned to have TCI had the target effect site concentration set at 3 μg mL^−1^ using the Schneider model and subsequently upward/downward titrated in 0.5 μg mL^−1^ increments to achieve and maintain a level of hypnosis measured by a BIS of 35–60. For patients who were assigned to have manual anesthesia induction, propofol was administered at a rate of 1.5–2.5 mg/kg to achieve the same level of hypnosis. Anesthesia was maintained by inhalation of an oxygen/air mixture (2/2 lt) with a 40% end-expiratory oxygen percentage and sevoflurane inhalation with a minimum alveolar concentration of 0.9–1.

In both groups, once an appropriate level of hypnosis was reached, 0.6 mg/kg rocuronium was administered to facilitate endotracheal intubation. Mechanical ventilation was started with a tidal volume of 8 mL/kg and a respiratory rate of 10–12 breaths/min. Hypnotics and opioids were titrated based on the patient’s hemodynamic response and BIS value (Covidien, Boulder, CO, USA). Post-induction hypotension was defined as a decrease in the mean arterial pressure (MAP) below 65 mmHg in the first 10 min after induction. If the MAP decreased below 65 mm/Hg, ephedrine was administered. 

### 2.6. Sets of Measurements

The duration of the measurements was defined from one minute before induction to 10 min after induction (before the surgical incision). After arrival in the operation room, radial artery cannulation was performed under local anesthesia, and the hemodynamic parameters began to be monitored with an uncalibrated pulse contour device (MostCare, Vytech, Vygon, Padova, Italy). Pre-induction, when the patient was still non-sedated, the baseline hemodynamic value was obtained by averaging three measurements taken while the patient was breathing deeply 6–8 times for one minute. Blood pressure and hemodynamic variables, such as the systolic arterial pressure (SAP), the mean arterial pressure (MAP), the diastolic arterial pressure (DAP), the heart rate (HR), the stroke volume variation (SVV), the pulse pressure variation (PPV), the arterial elastance (Ea), the cardiac power output (CPO), and the dp/dt were recorded at the same time points. The length of time required to reach a BIS below 60 was also noted as the induction time. 

All patients’ preoperative data, such as age, sex, weight, height, body mass index, comorbidities, use of beta-blockers and angiotensin-converting enzyme inhibitors, and physical status according to the ASA classification, were recorded in a virtual environment. 

The patient population was analyzed in two separate groups. First, the TCI and manual induction groups were compared, and second, patients who developed hypotension were compared with those who did not. 

### 2.7. Statistical Analysis

All data were reported as means (with standard deviations), medians (interquartiles), and percentages. The Kolmogorov–Smirnov test was used to test the normal distribution. Student’s t, Mann–Whitney U, and chi-square tests were used for comparisons between groups. To detect predictors of the risk of hypotension after anesthesia induction, a Cox regression analysis was used, to which all the significant parameters in the hypotension group were added. To obtain a 20% higher hypotension proportion in the manual induction group, the sample size was determined as 100 per group for β = 0.90 and ∝ = 0.05 (via an independent proportions test). *p* values < 0.05 were considered statistically significant. All statistical analyses were performed using SPSS version 29.0 (SPSS Inc., Chicago, IL, USA). 

## 3. Results

**Figure 1:**
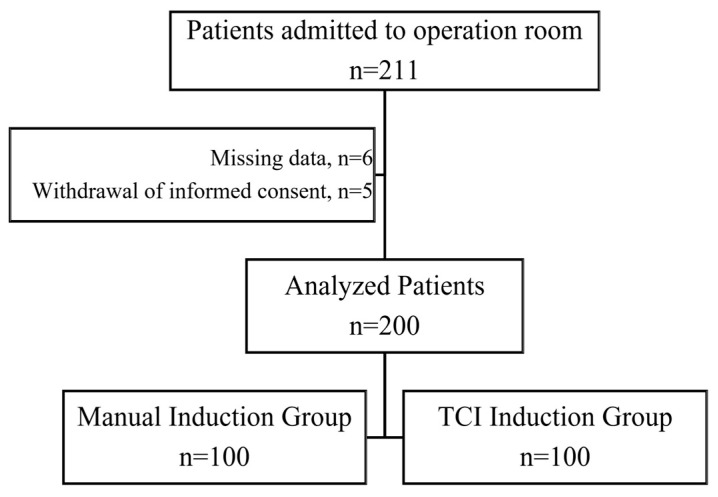
Flow diagram of the study participants.

**Figure 2:**
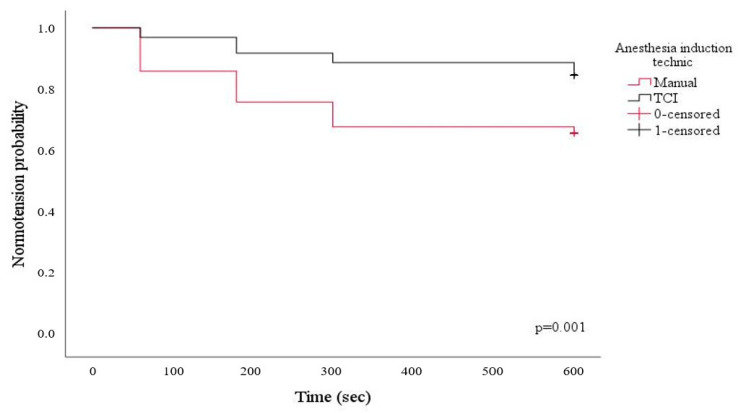
Kaplan–Meier curve of anesthesia induction methods for hypotension.

Of the 200 patients who completed the trial, 100 underwent anesthesia induction with TCI, and 100 underwent manual induction (Figure 1). The manual and TCI induction groups did not differ with respect to demographic data, ASA physical status, comorbidities, or preoperative medications (Table 1). Although the pre-anesthesia induction hemodynamic parameters were similar, post-induction hypotension was significantly higher in the manual induction group than in the TCI group (34% vs. 13%, respectively; *p* < 0.001). The Kaplan–Meier curve of anesthesia induction methods for hypotension is presented in Figure 2. After anesthesia induction, the CI, SVI, dp/dt, and CPO were significantly lower (*p* = 0.017, *p* = 0.004, *p* = 0.014, and *p* = 0.001, respectively), and the SVV (*p* < 0.001) and PPV (*p* < 0.001) were significantly higher in the manual induction group than in the TCI group. The comparison of the pre-and post-anesthesia induction hemodynamic parameters between the groups is presented in Table 2.

The propofol induction dose did not differ between groups (TCI: 155 (135–180) mg; manual: 150 (120–200) mg; *p* = 0.719), but the induction time (time from the start of propofol infusion/injection to a BIS < 60) was significantly longer in the TCI group than in the manual group (47 (35–60) s vs. 150 (105–220) s, respectively; *p* < 0.001).

Demographic data, ASA physical status, comorbidities (except hypertension), and preoperative medications were similar between the patients who developed hypotension and those who did not. Although similar percentages of both patient groups received antihypertensive treatment on the morning of the surgery, there were more hypertensive patients in the hypotension (+) group than in the hypotension (−) group (33 (70.2%) vs. 79 (51.6%) respectively; *p* = 0.029). The most common anesthesia induction method was manual induction in patients who developed hypotension. In the hypotension (+) group, the pre-induction SVI, CI, CPO, and dp/dt were significantly lower than in the hypotension (−) group (*p* < 0.001). Only the pre-induction Ea was higher in the hypotension (+) group (1.13 (0.98–1.50) mmHg m^−2^ mL^−1^ vs. 1.03 (0.86–1.17) mmHg m^−2^ mL^−1^, respectively; *p* = 0.006). A full comparison of the hypotension (+) and (−) groups is presented in Table 3.

In the multivariable Cox regression model, the risk of hypotension after anesthesia induction was increased 2.09-fold (1.06–4.08) by a history of hypertension, whereas a decreased risk of 0.93-fold (0.88–0.99), 0.30-fold (0.10–0.85), and 0.42-fold (0.21–0.84) was observed per unit decrease in SVI and CPO and the usage of the TCI as an anesthesia induction method, respectively (*p* = 0.032, *p* = 0.013, *p* = 0.024, and *p* = 0.015, respectively; Table 4).

## 4. Discussion

The present study aimed to evaluate the effectiveness of TCI induction with propofol in maintaining normotension after anesthesia induction and the ability of various hemodynamic parameters to predict hypotension in a patient population. The study’s results revealed that anesthesia induction with TCI was superior in maintaining normotension compared to the manual induction method. Additionally, the study found that the predictive abilities of PPV and SVV for hypotension were less sensitive in the patient population studied compared to other hemodynamic parameters, such as the CI, SVI, Ea, CPO, and dp/dt. Overall, these findings suggest that TCI may be an effective method for reducing post-induction hypotension risk, and the hemodynamic parameters monitored with PRAM, which shows cardiac performance, maybe a more reliable predictor of hypotension in this patient population.

Post-induction hypotension frequently develops, despite the knowledge of related risk factors, and is mainly caused by hypovolemia secondary to preoperative fasting and anesthetic agents such as propofol [2,13]. Certain risk factors, such as age, sex, weight, ASA score, and comorbidities associated with post-induction hypotension, have been described in various studies [2,14]. In the present study, the post-induction hypotension risk increased 2.09-fold (1.06–4.08) with a history of hypertension. It is generally not possible to change patient-related risk factors. Prophylactic measures, such as pre-induction volume replacement, also have limited effects on the prevention of post-induction hypotension [15]. Therefore, anesthetic agents and induction methods are important in reducing the risk of post-induction hypotension.

TCI is a computerized technique for administering intravenous drugs in anesthesia practice. It involves the use of a pharmacokinetic model of the drugs being administered, along with the patient’s individual characteristics, to achieve a predetermined concentration of the drug [16]. TCI can reduce individual variability by providing safer dosing with prompt titration of the drug using complex pharmacokinetic models that are not possible in manual infusions or bolus drug administration [9].

A Cochrane review by Leslie et al. compared computer-controlled administration with manual administration of propofol in terms of adverse events, such as hypotension, apnea, and movement during anesthesia [11]. Although TCI was associated with higher total doses of propofol, no difference was found in terms of adverse events. Due to the heterogeneity in the patient population and the use of different TCI models, this systematic review does not provide sufficient evidence.

Most studies comparing TCI and manual infusion groups have shown similar changes in blood pressure, HR, and hemodynamic instability occurrences [17,18,19]. Although a few studies have reported statistically significant differences in blood pressure or HR, these differences were generally not considered clinically significant [20,21]. Passot et al. reported a higher incidence of clinically significant hemodynamic instability in the manual group compared to the TCI group in a high-risk elderly patient population undergoing hip fracture surgery [22]. Their findings highlight that propofol administered through TCI offers cardiovascular stability, ensuring smooth induction and rapid recovery. In contrast, the use of manual-controlled infusion resulted in greater fluctuations in MAP, potentially leading to adverse effects. In the present study, the patients in the TCI group were also more stable with a lower rate of hypotension (*p* < 0.001). However, we should note that since most of our patients were relatively healthy with low ASA scores, it would be difficult to predict the results for a high-risk patient group. Chen et al. observed that similar induction and total doses of propofol were used and comparable hemodynamic stability was observed when anesthesia was induced and maintained with either a manual or a TCI system [23]. In this case, propofol administration was titrated to achieve a 40–60 BIS value (the same as in our study). In contrast, in the present study, we found that better hemodynamic stability was achieved in the TCI induction group, with similar induction doses and longer induction times than in the manual group. Nevertheless, administering propofol via a slow manual infusion during induction may yield comparable results to TCI. Consequently, conducting studies that compare manual anesthesia induction with different infusion rates to TCI anesthesia induction could be beneficial.

Predicting and identifying patients at risk of post-induction hypotension is as crucial as the induction method for the successful management of anesthesia. Various hemodynamic parameters, indexes, and tests have been utilized for the preoperative prediction of post-induction hypotension. Most studies have focused on patients’ volume status and fluid responsiveness and investigated the effects of the preoperative volume optimization. However, there are conflicting results regarding the predictive performance of the preload variables [24,25,26]. In the present study, the pre-induction hemodynamic parameters of SVI (*p* < 0.001), CI (*p* < 0.001), Ea (*p* = 0.006), CPO (*p* < 0.001), and dp/dt (*p* < 0.001) were significantly different between the groups compared to the hypotensive groups. However, the pre-induction preload indices, such as the SVV (*p* = 0.150) and PPV (*p* = 0.654), were similar between groups with and without hypotension.

Some studies report that patients are not hypovolemic even during long fasting periods and that pre-induction fluid optimization is not beneficial in preventing post-induction hypotension [15,27]. Therefore, evaluating the global cardiovascular performance of the patient rather than the volume status in the pre-induction period may provide more useful information to detect whether a patient is prone to post-induction hypotension. The present study demonstrated that cardiac performance is a more important determinant for identifying patients at risk for hypotension than preload variables, and in the multivariable Cox regression model, pre-anesthetic CPO and SVI were more sensitive to predicting post-induction hypotension. Patients with a lower CPO, dp/dt, CI, and SVI and a higher Ea are less able to tolerate the negative cardiovascular effect of propofol administration. 

The main cardiovascular effects of propofol are venodilation, arterial dilation, and a decrease in myocardial contractility [4,28]. However, it is not clear which hemodynamic mechanisms contribute the most to hypotension after anesthesia induction with propofol. The impact of propofol on CO is variable. While some studies have reported that propofol has minimal impact on CO, it has been reported to cause a significant decrease in others [29,30]. The effects of propofol on CO and the vascular system are dose- and time-dependent. Therefore, the net effect may vary from patient to patient, depending on the induction dose, duration, technique, and patient characteristics [7,31]. 

In the present study, although similar doses of propofol were administered in the manual and TCI induction groups, the induction time was longer in the TCI group, which also exhibited better preservation of cardiac performance parameters, such as the CI, dp/dt, and CPO, in the post-induction period compared to the manual group. The venodilation effect of propofol was probably more pronounced in the manual group, and the preload variables SVV and PPV were higher after induction than in the TCI group. However, we found that in both methods, Ea’s reflection of arterial tone and afterload were similar in the pre-induction period but did not change after induction. Overall, these results demonstrate that the anesthesia induction methods affect the cardiac system rather than the arterial system in this patient population.

This study had some limitations. Only one model (Schnider model) was used for TCI induction; the results may vary in other pharmacokinetic models. Furthermore, the study was conducted in a relatively healthy patient population with a low ASA score who underwent major abdominal surgery. The data do not provide information about the outcomes of patients with a higher ASA score. Finally, patients were free of severe pulmonary disease, cardiac valve dysfunction, and rhythm disorder, and these results cannot be extrapolated to the general population.

## 5. Conclusions

Our results show that TCI induction with propofol is superior to manual anesthesia induction with propofol in preventing post-induction hypotension. Furthermore, parameters reflecting cardiac performance and reserve, such as CPO and SVI, can predict post-induction hypotension more precisely than preload variables, such as PPV and SVV. Further studies on the use of various pharmacokinetic models in populations with higher ASA scores should be performed.

## Figures and Tables

**Table 1:** Demographic parameters between the manual induction and TCI induction groups.

Patients’ Characteristics	Manual Induction (n = 100)	TCI Induction (n = 100)	*p*
Age, years	64 (52–68)	61 (48–69)	0.717
Male, n (%)	69 (68.3)	58 (58.6)	0.153
BMI, (kg m^2^)	25.5 (23.5–28.9)	26.1 (23.4–29.8)	0.768
ASA score	2 (2–2)	2 (2–2)	0.183
Comorbidities, n (%)			
Hypertension	56 (55.4)	56 (56.6)	0.873
Diabetes mellitus	26 (25.7)	20 (20.2)	0.352
COPD	9 (8.9)	11 (11.1)	0.644
CHF	3 (3.0)	1 (1.0)	0.621
CAD	20 (19.8)	14 (14.1)	0.287
CVD	3 (3.0)	3 (3.0)	0.649
CRF	3 (3.0)	1 (1.0)	0.621
Preoperative medications, n (%)			
B-blocker	17 (16.8)	20 (20.2)	0.539
ACE inhibitors	5 (5.0)	5 (5.1)	1.000

BMI, body mass index; ASA, American Society of Anesthesiologists; CAD, coronary artery disease; CHF, chronic heart failure; COPD, chronic obstructive pulmonary disease; CRF, chronic renal failure; CVD, cerebrovascular disease.

**Table 2:** Comparison of pre- and post-anesthesia induction hemodynamic parameters between the manual induction and TCI groups.

	Pre-Anesthesia Induction			Post-Anesthesia Induction		
	Manual Induction (n = 100)	TCI Induction (n = 100)	*p*	Manual Induction (n = 100)	TCI Induction (n = 100)	*p*
HR, min^−1^	73 (66–82)	72 (65–85)	0.951	72 ± 13	68 ± 11	*0.044*
SAP, mmHg	144 ± 22	147 ± 18	0.321	108 (88–121)	118 (99–138)	*0.001*
DAP, mmHg	70 ± 9	72 ± 9	0.175	58 (50–67)	64 (56–70)	*<0.001*
MAP, mmHg	94 ± 13	97 ± 11	0.131	75 ± 14	81 ± 13	*<0.001*
SVI, mL/m^2^	43 (37–51)	46 (40–54)	0.112	36 ± 11	40 ± 11	*0.004*
CI, L/min/m^2^	3.1 (2.7–3.9)	3.4 (2.9–3.9)	0.107	2.4 (2.0–2.8)	2.5 (2.3–3.0)	*0.017*
SVV, %	15 (12–17)	15 (13–16)	0.400	12 (8–14)	8 (5–10)	*<0.001*
PPV, %	15 (13–16)	15 (14–16)	0.204	12 (9–16)	8 (6–12)	*<0.001*
Ea, mmHg m^−2^ mL^−1^	1.08 (0.86–1.28)	1.05 (0.90–1.18)	0.531	1.07 (0.91–1.34)	1.07 (0.91–1.27)	0.837
CPO, watt	1.23 (1.03–1.66)	1.40 (1.08–1.78)	0.120	0.74 (0.57–0.97)	0.81 (0.71–1.13)	*0.001*
dp/dt, mmHg/msn	1.33 (1.10–1.70)	1.32 (1.13–1.70)	0.624	0.80 (0.60–1.01)	0.90 (0.71–1.14)	*0.014*
Induction propofol dose, mg				150 (120–200)	155 (135–180)	0.719
Duration of BIS < 60, sec				47 (35–60)	150 (105–220)	*<0.001*
Hypotension after induction n (%)				34 (33.7)	13 (13.1)	*<0.001*
Epinephrine dosage, mg				0 (0–10)	0 (0–5)	0.362

HR, heart rate; SAP, systolic arterial pressure; DAP, diastolic arterial pressure; MAP, mean arterial pressure; SVI, stroke volume index; CI, cardiac index; SVV, stroke volume variation; PPV, pulse pressure variation; Ea, arterial elastance; CPO, cardiac power output, BIS, bispectral index.

**Table 3:** Comparison between patients with hypotension (−) and (+).

Patients’ Characteristics	Hypotension (−) (n = 153)	Hypotension (+) (n = 47)	*p*
Age, years	62 (48–69)	64 (55–68)	0.583
Male, n (%)	97 (63.4)	30 (63.8)	0.957
BMI, (kg m^2^)	26.0 (23.3–30.1)	25.5 (23.9–27.8)	0.452
ASA score	2 (2–2)	2 (2–2)	0.183
Comorbidities, n (%)			
Hypertension	79 (51.6)	33 (70.2)	*0.029*
Diabetes mellitus	37 (24.2)	9 (19.1)	0.555
COPD	15 (9.8)	5 (10.6)	0.789
CHF	2 (1.3)	2 (4.3)	0.235
CAD	26 (17.0)	8 (17.9)	1.000
CVD	5 (3.3)	1 (2.1)	1.000
CRF	4 (2.6)	0 (0.0)	0.575
Preoperative medications, n (%)			
B-blocker	28 (18.3)	9 (19.1)	1.000
ACE inhibitors	6 (3.9)	4 (8.5)	0.249
Pre-anesthesia induction			
HR, min^−1^	72 (65–81)	78 (68–85)	0.088
SAP, mmHg	147 ± 17	142 ± 23	0.176
DAP, mmHg	72 ± 8	70 ± 10	0.233
MAP, mmHg	98 ± 10	94 ± 14	0.124
SVI, mL/m^2^	48 (41–56)	39 (33–45)	*<0.001*
CI, L/min/m^2^	3.3 (2.9–4.1)	2.9 (2.6–3.2)	*<0.001*
SVV, %	15 (12–16)	15 (13–18)	0.150
PPV, %	15 (14–16)	15 (14–16)	0.654
Ea, mmHg m^−2^ mL^−1^	1.03 (0.86–1.17)	1.13 (0.98–1.50)	*0.006*
CPO, watt	1.42 (1.15–1.81)	1.03 (0.82–1.30)	*<0.001*
dp/dt, mmHg/msn	1.40 (1.19–1.80)	1.13 (0.94–1.40)	*<0.001*
Anesthesia induction technique, n (%)			
Manual induction TCI induction	67 (43.8) 86 (56.2)	34 (72.3) 13 (27.7)	*<0.001*
Epinephrine dosage, mg	0 (0–0)	15 (10–20)	*<0.001*

BMI, body mass index; ASA, American Society of Anesthesiologists; CAD, coronary artery disease; CHF, chronic heart failure; COPD, chronic obstructive pulmonary disease; CRF, chronic renal failure; CVD, cerebrovascular disease HR, heart rate; SAP, systolic arterial pressure; DAP, diastolic arterial pressure; MAP, mean arterial pressure; SVI, stroke volume index; CI, cardiac index; SVV, stroke volume variation; PPV, pulse pressure variation; Ea, arterial elastance; CPO, cardiac power output; TCI, target-controlled infusion.

**Table 4:** Univariable and multivariable Cox regression analysis for the risk of hypotension.

	Univariable		Multivariable	
	HR (95% CI)	*p*	HR (95% CI)	*p*
Hypertension	2.2 (1.2–4.2)	*0.012*	2.09 (1.06–4.08)	*0.032*
Pre-anesthesia induction parameters				
SVI	0.96 (0.93–0.98)	*<0.001*	0.93 (0.88–0.99)	*0.013*
CPO	0.24 (0.11–0.50)	*<0.001*	0.30 (0.10–0.85)	*0.024*
CI	0.55 (0.37–0.81)	*0.003*	1.97 (0.89–4.36)	0.096
Ea	2.20 (1.00–4.80)	*0.045*	0.46 (0.12–1.81)	0.267
dp/dt	0.33 (0.15–0.74)	*0.007*	1.07 (0.41–2.80)	0.897
The usage of the TCI	0.38 (0.20–0.72)	*0.003*	0.42 (0.21–0.84)	*0.015*

CI, cardiac index; CI, confidence interval; CPO, cardiac power output; Ea, arterial elastance; HR, hazard ratio; TCI, target-controlled infusion. Enter method was used in the multivariate cox regression. Omnibus test significance: *p* < 0.001.

## Data Availability

The datasets for the current study are available from the corresponding author upon reasonable request.
